# Work-related stress amongst doctors in intensive care, anaesthetics, accident and emergency and general medicine

**DOI:** 10.1186/cc12446

**Published:** 2013-03-19

**Authors:** JI Tuthill, MS Ahmed, G Mathew, AC Bolton, AA Molokhia

**Affiliations:** 1 University Hospital Lewisham, London, UK

## Introduction

Work-related stress is a potential problem among doctors and is associated with anxiety, depression, reduced job satisfaction, days off work, errors and near misses [1]. To compare stress levels between different groups of doctors and identify causes of stress, we conducted a survey at University Hospital Lewisham using the UK Health and Safety Executive's Management Standards (HSEMS). HSEMS is a validated tool developed to identify work conditions that warrant interventions to reduce stress levels across organisations [2].

## Methods

We conducted an anonymous survey of doctors working in anaesthetics, intensive care, general medicine and accident and emergency (A&E) departments over 6 weeks using the HSEMS questionnaire. We also surveyed awareness of the Trust's stress management services and whether staff had a designated supervisor or mentor. Results were analysed using the HSEMS Analysis Tool, which rates stressors with a score from 1 to 5 (5 represents the lowest amount of stress). We compared the Trust's results against HSEMS national standards.

## Results

Seventy-two doctors completed the survey. Lowest stress levels were found in doctors working in intensive care (*n *= 12, mean 3.63, SD 0.39). This was followed by medicine (*n *= 26, mean 3.55, SD 0.47), anaesthetics (*n *= 27, mean 3.40, SD 0.44), and A&E (*n *= 7, mean 3.11, SD 0.65), which had the highest stress levels. There was no significant difference in stress levels between different grades of doctors. When compared with HSEMS targets, staff relationships and peer support exceeded national standards. However, management of organisational change and demands at work need improvement. The majority of doctors (82%) had no idea what stress management services were provided by the Trust. Seventy-nine per cent of doctors had an allocated supervisor or mentor, 91% of those felt able to approach their supervisor.

## Conclusion

These survey results provide reassurance that stress levels in intensive care compare well, despite critically unwell patients and higher mortality rates. We identified areas that need improvement within the Trust and will present these results to all relevant departments. With the support of hospital management we will initiate HSEMS-validated measures to reduce stress.
